# Victim's Perspective of Forgiveness Seeking Behaviors After Transgressions

**DOI:** 10.3389/fpsyg.2021.656689

**Published:** 2021-04-21

**Authors:** Pilar Martinez-Diaz, Jose M. Caperos, Maria Prieto-Ursúa, Elena Gismero-González, Virginia Cagigal, Maria José Carrasco

**Affiliations:** ^1^Department of Psychology, Comillas Pontifical University, Madrid, Spain; ^2^UNINPSI Clinical Psychology Center, Comillas Pontifical University, Madrid, Spain; ^3^Fundación San Juan de Dios, Madrid, Spain

**Keywords:** forgiveness, forgiveness seeking, transgression, close relationships, relational repair, restoration, apologies, accounts

## Abstract

Forgiveness seeking after a relational transgression is an important aspect of relational repair from an interpersonal perspective, although it has received much less attention than the process of granting forgiveness. This research focuses on the victim's perspective of the transgressor's behaviors and how they are related to forgiveness and offense characteristics. This paper proposes a multidimensional concept of seeking forgiveness that includes four dimensions: apologies, restorative action, relational caring behaviors, and diverting behaviors. A questionnaire for assessing these dimensions was developed and tested with a general population sample of 450 subjects. Participants recalled a specific offense and then answered a questionnaire about the perceived usefulness of different forgiveness-seeking behaviors, a forgiveness inventory, and several questions regarding the characteristics of the offense (severity, intentionality, and frequency). Our results support the four-factor structure of the questionnaire. As the perceived intentionality of the offense increases, behaviors that are directly related to the transgression, such as apologies and restorative actions, are experienced as less useful for forgiveness. The more hurtful the offense, the less useful the diverting behaviors are. Behavior such as apologies and restorative action are related to a lower (less) motivation for revenge, while all forgiveness-seeking behaviors are related to an increase in feelings of benevolence toward the offender.

## Introduction

Close relationships are built on trust, affection, and intimacy. They are the source of deeply gratifying experiences, but also a place where one can be hurt, deceived, or offended. Of particular significance are relational transgressions, that is, when a partner's behavior violates the explicit or implicit relational rules, with the accompanying emotions of anger, sadness, and withdrawal, and the resulting deterioration of the relationship (Cordova et al., 2006; Metts and Cupach, 2007). In this context, forgiveness, defined as the process by which negative emotions (such as revenge, avoidance, or blame) diminish and are replaced by benevolence (McCullough et al., 1998; McCullough, 2000), has been found to be a predictor of pro-relationship motivation (Karremans and Van Lange, 2004) and a key ingredient in the maintenance and repair of interpersonal relations (Fincham et al., 2006). From an interpersonal perspective, the perpetrator's behavior after the offense affects the probability of forgiveness and relationship repair, as well as the quality of the post-offense relationship (Fincham et al., 2006; Hannon et al., 2010). The attitudes and behaviors of the offender play a vital role in lessening blame, avoidance, and desire for revenge, replacing these feelings with goodwill, and helping to restore trust (Rusbult et al., 2005).

However, research on the dark side of forgiveness shows that sometimes forgiving may seem counter-effective, as it may increase the likelihood of future negative behavior by the offender or lead to a decrease in the victim's self-concept and self-respect (Luchies et al., 2010; McNulty, 2011, 2020). McNulty (2008, 2011) found that forgiveness increases the likelihood of negative behavior (physical and verbal aggression) in distressed couples and can be harmful over time. In the same line, Luchies et al. (2010) determined that when the perpetrator signals that the victim will be safe in the relationship by making sincere amends, the victim's self-concept and self-respect are bolstered. However, if the perpetrator does not offer amends, the victim's self-concept and self-respect might be diminished. Hence, whether forgiveness has a positive or negative effect on the victim is closely related to the offender's motivation and post-offense behavior. The results of this research beckon a detailed examination of the processes and factors that allow for healthy outcomes of the forgiveness process.

Previous research has focused mostly on the phenomenon of granting forgiveness, while the act of seeking forgiveness has received much less attention (Kelley and Waldron, 2005; Bassett et al., 2006). The number of studies on forgiveness-seeking behaviors after a relational transgression is increasing, but a better understanding of the process is needed. Some theories have addressed behavioral strategies that individuals use to heal the hurt and repair a relationship after a transgression (Merolla, 2008; Waldron and Kelley, 2008; Morse and Metts, 2011). However, these approaches do not always agree on the components of its dimensions; e.g., some studies subsume actions that reflect relationship caring rituals in an independent dimension (Aune et al., 1998; Pansera, 2009), but most do not take these strategies into account (e.g., Morse and Metts, 2011) or they consider them as areas of other dimensions (e.g., Kelley and Waldron, 2005; Rourke, 2007). Moreover, most studies have focused mostly on one pattern of action—apologies—and do not consider others such as restorative behaviors, which have been found to be useful in repairing a relationship transgression (Hargrave, 1994; Dindia, 2003). Finally, few models consider strategies in which there is not an acknowledgment of the damage and responsibility and, thus, do not show genuine repentance and seeking of forgiveness. These behaviors are more motivated by a defensive need to maintain one's image and to resume the relationship without genuine forgiveness and repair (McLaughlin et al., 1983; Woodyatt and Wenzel, 2013b; Schumann and Orehek, 2017).

The aims of this study are to describe the transgressor's main forgiveness-seeking strategies that have been identified in the literature on interpersonal forgiveness, to develop a new questionnaire for assessing to what extent these behaviors are useful for the victim in order to grant forgiveness.

### An Approach to Forgiveness Seeking

Based on a review of the literature Waldron and Kelley (2008) propose a theoretical model of the forgiveness process from an interpersonal perspective comprising four components. The first element is the nature of the relationship before the offense. The process and negotiation of forgiveness depends on the type of relationship (work, friends, and family), the relationship quality and length at the time of the offense. The second is motivation, that is the reasons that drive the person to seek forgiveness and the personal characteristics related with granting of forgiveness. The third component is the communication behaviors through which individuals manage their reactions to the offense, and how they seek and grant forgiveness. Finally, the fourth component refers to the relational outcomes of the forgiveness process.

There is an interplay of these components to understanding how the complex process of forgiveness from an interpersonal perspective unfolds. In this research, we focus on the third component, the seeking of forgiveness, analyzing the importance for the victim of certain transgressor's behaviors, that is, to what extent these behaviors are clues indicating to the victim that it is adequate to forgive high severity offenses. The transgressor's behavior as part of the communication of forgiveness is related with the post-transgression relational outcomes (Kelley and Waldron, 2005; Merolla and Zhang, 2011).

According to the theoretical framework developed by Gordon et al. (2004) for the intervention with severe transgressions in committed relationships, three tasks have to be accomplished in the forgiveness process in order to achieve positive relational outcomes (Gordon and Baucomb, 2003; Gordon et al., 2004): absorbing the emotional impact of the event; understanding and reconstructing the relationship rules and the third stage in which the negative affect decreases and they move beyond the offense and sometimes negotiate their “relational covenant” (Hargrave, 1994). Throughout the process of forgiveness, these types of behaviors displayed by the offender give the victim a clue as to whether the transgressor has engaged in a genuine process of forgiveness (acknowledging the harm, making amends) or, if that is not the case, and it might be unsafe to forgive (Gordon et al., 2020).

Our first step for studying forgiveness seeking behaviors and their role in the forgiveness process was to identify them through a review of the two main theoretical approaches in the study of relational repair after a transgression: those based on forgiveness theory and those grounded in interpersonal communication theory. The review of research on repair behaviors from both approaches enables us to identify two types of actions that might be used when seeking forgiveness: remedial behaviors and repair strategies.

*Remedial behaviors* are behaviors that the offender may use to explain the transgression so that it becomes more understandable and the relationship can continue (Morse and Metts, 2011). Research on this area mainly addresses one type of forgiveness-seeking behavior: *offering apologies* (Fehr and Gelfand, 2010; Lewis et al., 2015). Lazare (2004) considered that a good apology restores the victim's dignity and self-respect, which are damaged by the transgression, and helps victims to have a more acceptable view of the offender, depending on the extent to which the offender communicates feelings of sorrow or suffering for the harm caused. Other remedial actions that have been studied less extensively are *accounts*, which aim to reduce the offender's responsibility for the transgression through behaviors such as excuses, justifications, or denial. The effectiveness of these strategies at restoring trust varies, being denial the least effective strategy (Gracyalny et al., 2008; Morse and Metts, 2011). These strategies are not accompanied on the part of the aggressor with a genuine forgiveness process and hinder forgiveness and relational repair (Hodgins and Liebeskind, 2003; Woodyatt and Wenzel, 2013a).

*Repair strategies* involve an actual change in the offender's behavior in an attempt to seek forgiveness. Such behaviors have been analyzed using the accommodation model proposed by Rusbult et al. (1991). This model classifies repair behaviors on a continuum from active to passive. According to the summary presented by Guerrero et al. (2014), two main constructive-active strategies can be identified: (1) prosocial communication, and (2) discussion and problem solving. Prosocial communication refers to behaviors that “focus more on reestablishing closeness and connection rather than solving problems” (p. 360), that is, behaviors that diminish discomfort through affection and promoting closeness (Waldron and Kelley, 2008) and are related with relational maintenance behavior (Dindia, 2003). The second, discussion and problem solving, covers a range of actions such as modifying behaviors that are directly related to the transgression and has been studied in research on forgiveness seeking under the terms of compensation, amends, or restorative action.

### Main Dimensions of Forgiveness Seeking: A Proposal

Taking into account the review of literature on forgiveness-seeking, we conceptualize forgiveness seeking as a set of behaviors aimed at reducing negative emotions in the victim and promoting closeness (Hargrave, 1994; Dindia, 2003; Kelley and Waldron, 2005; Pansera, 2009; Morse and Metts, 2011; Lewis et al., 2015). Like other authors, we consider this concept from a multidimensional perspective (Sandage et al., 2000; Bassett et al., 2006) and propose four main dimensions of forgiveness-seeking behaviors: apologies, restorative action, relational caring, and diverting strategies.

#### Apologies

Apologies are the most common and effective forgiveness-seeking behaviors, and the most widely studied in the field of forgiveness-seeking (McCullough et al., 1998; Zechmeister et al., 2004; Bachman and Guerrero, 2006). The offer of an apology consistently emerges as a predictor of forgiveness: this behavior reduces the negative effects of the transgression and thus facilitates forgiveness and increases the possibility of relational repair (Emmers and Canary, 1996; Kelley, 1998; McCullough et al., 1998; Hodgins and Liebeskind, 2003; Bachman and Guerrero, 2006; Morse and Metts, 2011). Apologies are effective if they are perceived to be sincere and include an admission of guilt (Zechmeister et al., 2004; McCullough, 2008; Slocum et al., 2011b).

Several psychological mechanisms have been described that underlie the effectiveness of apologies (McCullough et al., 1998; Davis and Gold, 2011; Carlisle et al., 2012). From a motivational perspective, they increase the victim's empathy toward the transgressor, particularly if a close relationship exists between them (McCullough et al., 1998). Another mechanism is related to an attribution model: apologies change the attribution of responsibility and/or decrease the attribution of behavioral stability, leading to a perception that the offense will not happen again (Davis and Gold, 2011) and restoring the victim's self-respect and dignity (Lazare, 2004; McCullough, 2008). Since apologies are costly for the offender, they make up for some of the injustice and restore the balance and equity between victim and offender (Exline et al., 2003; Carlisle et al., 2012).

Nevertheless, apologies do not always increase the probability of forgiveness being granted. Apologies are less helpful for granting forgiveness when the transgression is repeated, or the victim expects that it will be repeated in the future (Gold and Weiner, 2000; Davis and Gold, 2011; Morse and Metts, 2011), when transgressions are more severe (Ohbuchi et al., 1989; Merolla, 2008; Pansera, 2012), and when the offense is perceived to be intentional (Struthers et al., 2008).

Based on Ashby's (2003) definition, we consider that a full offer of apology encompasses three components: admitting responsibility for the untoward action, acknowledging the pain that has been inflicted, and expressing remorse and repentance. Following other authors (Fehr and Gelfand, 2010; Slocum et al., 2011a; Lewis et al., 2015) verbal offers of compensation are included in this dimension but specific changes in behavior will be addressed as part of *Restorative action*.

#### Restorative Action

This is an essential part of the forgiveness process, as it helps to promote emotional repair (Hargrave, 1994; Ashby, 2003; McCullough, 2008; Slocum et al., 2011a; Lewis et al., 2015). A restorative action is related to a change in behavior and/or the rules of the relationship in relation to the offense, which increases the psychological security that the event will not be repeated (Carlisle et al., 2012). Hargrave's model of forgiveness in families establishes that a necessary phase of the process is the need for compensation and should imply some form of change that is balanced and trustworthy (Hargrave, 1994). To decide whether it is safe to continue the relationship, the offended person must be able to evaluate the offender's readiness to treat him/her in a different way (Hargrave and Zasowski, 2017). Acts of compensation that involve behavior changes undo some of the damage caused by the transgression, increase trust, and seem to provide the certainty that the offense will not happen again (Exline and Baumeister, 2001; McCullough, 2008; Carlisle et al., 2012; Hargrave and Zasowski, 2017).

Specific restorative actions and more elaborate amends become necessary the more severe the offense (Pansera, 2009; Merolla and Zhang, 2011), when there is an increased perception of responsibility or blameworthiness (Merolla, 2008; Merolla and Zhang, 2011), and when relationship satisfaction is lower (Pansera, 2009). Severe transgressions provoke greater suffering and distress and increase doubts about the safety of the relationship and the partner's future behavior (Afifi and Metts, 1998; Pansera, 2009). Furthermore, in high-blame offenses that are attributed to the offender's characteristics and when the probability of reoffending is perceived to be high, the victim tends to grant conditional forgiveness as a way of ensuring against further misdeeds and of enacting justice (Merolla and Zhang, 2011; Kloeber and Waldron, 2017; Sheldon and Antony, 2018) and, thus, the agressor's restorative action will be more valued.

In our proposal, restitution is characterized by a restorative action. Here, behaviors that seek to right the wrong or steps to prevent the re-occurrence of the offense come into play. These behaviors are similar to what Hargrave (1994) called change in the “relationship covenant,” and to what has been called *negotiated forgiveness*, where the offended party sets conditions as part of the forgiveness process (Andrews, 2000; Waldron and Kelley, 2008). This dimension does not include verbal promises of change: it focuses on specific behavior change carried out by the offender.

#### Relational Caring Behaviors

These are verbal and nonverbal behaviors that do not address the offense directly but are undertaken to demonstrate that the offender cares and hopes to reconnect with the victim of the offense (Dindia, 2003; Pansera, 2009), such as being more attentive, spending more time with the partner, or giving presents. These behaviors have been associated with forgiveness, positive relational outcomes and reconciliation (Aune et al., 1998; Gracyalny et al., 2008). Most authors highlight the close link between these actions and the recovery of a satisfactory relationship after the transgression (Dindia, 2003; Pansera, 2009), although few studies have explored the association between relational caring behaviors and forgiveness.

We consider that relational caring behaviors play a vital role in promoting reconciliation between the victim and the offender and reduce rejection and mistrust. These behaviors are essential in the routine maintenance of a relationship. Caring behaviors are related to prosocial and ceremonial strategies in Dindia and Baxter's typology of maintenance and repair strategies (Dindia and Baxter, 1987; Dindia, 2003).

#### Diverting Strategies

A key behavior for a genuine process of forgiveness is the transgressor's acceptance of responsibility (Wohl and McLaughlin, 2014). However, as this acceptance requires transgressors to admit personal failure and recognize the harm caused by their actions, they may instead choose to protect their image and reduce emotional distress through defensive strategies (Schumann and Orehek, 2017). These defensive strategies are also called pseudo self-forgiveness and involve minimization of harm and denial of wrongdoing and its hurtful effect on the victim (Woodyatt and Wenzel, 2013a).

There are several classifications of defensive strategies which vary according to the degree to which aggressors accept the threat to their self-image, responsibility and guilt (McLaughlin et al., 1983; Hodgins and Liebeskind, 2003). For the purpose of this research we have identified the ones most commonly reported in the literature: excuses, justification and avoidance. In e*xcuses*, the offender admits that the offense has occurred, but minimizes responsibility for it and refers to some external cause (McLaughlin et al., 1983; Hodgins and Liebeskind, 2003; Rourke, 2007); *justification*, in which the offender admits responsibility for the act, but denies that it was an offense and/or minimizes its importance (McLaughlin et al., 1983; Ferrara and Levine, 2009); and *avoidance*, in which the offender evades or refuses to talk about the issue (McLaughlin et al., 1983; Aune et al., 1998; Hodgins and Liebeskind, 2003; Metts and Cupach, 2007; Sheldon and Antony, 2018).

From the perspective of the transgressor, diverting strategies are indicators of an attempt to avoid assumption of responsibility and a tendency to self-protection and are related with avoidant attachment styles (Hodgins and Liebeskind, 2003; Schumann and Orehek, 2017). Several studies have analyzed the use of diverting behaviors to repair a relationship breach (Aune et al., 1998; Rourke, 2007; Morse and Metts, 2011) and its relation with relational outcomes. The transgressor's report of defensive responses has been related with worse interpersonal outcomes such as less empathy for the victim and less desire for reconciliation (Woodyatt and Wenzel, 2013a), less expectation of a positive relationship (Hodgins and Liebeskind, 2003) and related with transgressor whose aim is more own face repair than relationship repair (Hodgins and Liebeskind, 2003; Schumann and Orehek, 2017).

However, from the victim's perspective, there have been few studies focused on the effect of these strategies on the victim's forgiveness process, and those that have been carried out provide an interesting perspective regarding these strategies. For example, Aune et al. (1998) found that excuses, justifications and avoidance were not related to worse relational outcomes, and Ohbuchi and Sato (2001) found that children accept excuses and tend to forgive when there is low attribution of responsibility. These findings may indicate that for victims who tend to forgive without the aggressor showing signs of true repentance, these strategies might be considered a viable option and serve as a way of dealing with deception and minimizing conflict (Lewis et al., 2015). Defensive strategies are more acceptable when there is low commitment. For example, Kelley (1998) found that humor was used to seek forgiveness, but it was rarely used by romantic partners, perhaps because it might be perceived as a failure to recognize the harm caused (Kelley and Waldron, 2005). Also, Sheldon and Antony (2018) found that dating couples use more minimizing strategies than married couples, indicating that it is less common in stable relationships.

Diverting behaviors are included in our approach to forgiveness seeking, as they can be associated with forgiveness. They could placate the victim and provide a more acceptable image of the offender. However, they do not necessarily imply a change of behavior and are, therefore, not related to reestablishing a quality relationship.

Regarding the link between forgiveness-seeking behaviors and the tasks of the forgiveness model, it seems plausible that apologies are required, particularly in the initial stage, to absorb the emotional impact and make sense of the situation, since in the emotional turmoil generated by the transgression, the victim needs to sense that the offender acknowledges the pain caused and accepts responsibility for the action. Caring behaviors and restorative action will be more needed after the emotional impact decreases, and will more probably help to increase the sense of safety and security (McCullough, 2008; Merolla and Zhang, 2011) opening the door to creating a new understanding of the relationship, if the relationship continues. These three forms of searching forgiveness could indicate that the transgressor has gone through a process of intrapersonal forgiveness. Nevertheless, the transgressor sometimes engages in defensive strategies in order to protect the self and shifts the blame to external causes or minimizes the impact of the offense on the victim (Wohl and McLaughlin, 2014). In this case, the forgiveness-seeking behaviors most likely to increase would be diverting strategies.

### Instruments to Assess Forgiveness Seeking

Several instruments have been published for assessing relational repair strategies (Aune et al., 1998; Morse and Metts, 2011) or forgiveness-seeking strategies (Sandage et al., 2000; Kelley and Waldron, 2005; Bassett et al., 2006; Rourke, 2007; Chiaramello et al., 2008; Pansera, 2009; Pansera and La Guardia, 2012). Two of these instruments (Bassett et al., 2006; Chiaramello et al., 2008) assess motivation or disposition to seek forgiveness rather than the behaviors that the offender displays to achieve it and, therefore, will not be considered in this section.

The other six questionnaires that assess actions the offender takes to obtain forgiveness can be classified according to whether they take the perspective of the victim or that of the offender. Although both parties play important roles in the forgiveness process, we examine the victim's perspective to establish the impact that the offender's behavior has on the victim and, therefore, under which circumstances forgiveness is more likely and more adequate to be granted. Three of the instruments above take the victim's perspective into account (Kelley and Waldron, 2005; Morse and Metts, 2011; Pansera and La Guardia, 2012).

However, according to the theoretical guidelines outlined in the previous section which highlight the importance of considering both constructive and defensive strategies when seeking forgiveness and to differentiate the dimensions, we consider that a forgiveness-seeking questionnaire that takes into account the victim's perspective should meet the following criteria.

First, it should cover the plurality of behaviors with which the offender can seek forgiveness and include forgiveness seeking guided by the acceptance of moral responsibility and a genuine attempt at interpersonal reparation, as well as pseudo-forgiveness process behaviors in which the transgressor uses some defensive strategy to minimize their responsibility, or the harm they committed. Secondly, the different forgiveness-seeking strategies should be assessed in separate dimensions, with the various behaviors associated with it being assessed more thoroughly and measured independently, as these can have a differential importance in the forgiveness process depending on the contextual factors (e.g., type and quality of relationship, characteristics of the offense).

Finally, within each of the dimensions indicated in the theoretical review, the items should cover the main aspects of each dimension. For example, apologies should include both the recognition of responsibility and the recognition of the damage caused; and repair behaviors must include, in addition to verbal responses of intention to change, other statements that indicate specific repair and change behaviors.

Among the existing forgiveness-seeking questionnaires, none of them met all the criteria set out above and therefore a new questionnaire was developed that meets these characteristics and does it from the victim's perspective.

### Transgression Characteristics

The characteristics of the offense impact how forgiveness unfolds and the value that the victim gives to the post-transgression behaviors. Among these characteristics, the review of the literature highlights three: severity, intent, and frequency of the offense.

#### Severity

Severity of the transgression is related to the norms and expectations of a given relationship and therefore, the more severe the transgression, less probability of forgiveness (Fincham and Jackson, 2005; Rusbult et al., 2005; Morse and Metts, 2011) and more actions will be needed to repair the relationship. Indeed, research shows that for severe transgressions, the intensive use of all the forgiveness tactics was related with forgiveness Kelley and Waldron (2005) and more frequently is related with conditional forgiveness (Merolla, 2008; Struthers et al., 2008).

#### Intentionality

One of the first responses of the victim is to assess whether the offense was an unintentional mistake, or an intentional action. Individuals are less forgiving of offenses that are perceived as intentional (Fincham and Jackson, 2005; Waldron, 2005). Intentional offenses elicit more anger, and the victims expect more to receive an apology than in unintentional offenses (Green et al., 2020), although apologizing might not be enough to be forgiven (Struthers et al., 2008). In instances, when there is an attribution that the offender intended the negative outcome, it is harder to forgive and more restorative action together with strong apologies are needed (Ohbuchi et al., 1989; Fincham and Jackson, 2005).

#### Frequency

Research shows that severe transgressions are easier to forgiven when they are isolated incidents (Gunderson and Ferrari, 2008). Additionally, offense frequency is usually related with severity with more frequent offenses being considered more severe.

In summary, given the importance of repairing interpersonal relationships after a transgression, particularly in ongoing relationships, we considered that it will be useful in the study of forgiveness seeking to devise a questionnaire for assessing the victim's perception of the usefulness of different forgiveness seeking strategies for granting forgiveness. These strategies include acknowledging the hurt and taking steps to avoid it from happening again, behaviors intended to care for the relationship, and a fourth category of strategies that amend the offender's image by reframing the meaning and importance of the transgression. We expected to find a four factor structure that resembles the four proposed dimensions: apologies, restorative action, caring behaviors and diverting strategies.

We expected to find that the greater the severity of the offense, its perceived intentionality, and frequency, the more useful specific restorative actions will be to gain forgiveness. As the literature review suggests, when the victim perceives that the severity and responsibility are high, more elaborate apologies and amends are needed to gain forgiveness (Merolla, 2008; Merolla and Zhang, 2011). Under these circumstances, diverting behaviors will be perceived as less useful.

## Materials and Methods

### Procedure and Participants

We used a non-probabilistic convenience sampling method contacting the participants through colleagues, community leaders and acquaintances who handed the questionnaires along with a prepaid return envelope. The subjects received a packet containing brief instructions, an informed consent form, the questionnaires, and a pre-paid self-addressed return envelope for their response.

The set of questionnaires included some demographic data and instructions to recall a specific offense in which someone had hurt them deeply and unjustly. From the perspective of that specific offense, they had to respond to the type of relationship with the offender, the characteristics of the offense, the offender's forgiveness seeking and relational repair behaviors, and the degree of forgiveness experienced^1^.

Participants were 450 Spanish adults recruited by nonprobability sampling. Of these, 293 (65.1%) were women, with a mean age of 37.4 (SD = 15.4) ranging from 18 to 80 years old. A total of 45.6% of the subjects were working, 38.5% were students, and the remaining 16.0% were unemployed, retired, or were active homemakers. In terms of educational level, 70.5% have completed university studies, 25.0% have completed high school studies, and 4.5% have not finished high school. The offender was a friend of the victim in 33.0% of the cases, a family member in 18.8%, a partner in 19.6%, a coworker in 17.4%, and otherwise identified in 11.2% of the cases.

#### Characteristics of the Offense

Mean severity of the offense was 3.24 (SD = 1.08; MIN = 1; MAX = 5) intentionality mean = 2.93 (SD = 1.28; MIN = 1; MAX = 5) and frequency mean = 1.96 (SD = 0.93; MIN = 1; MAX = 4). Time from the offense was positively related to severity, *r* = 0.186; *p* < 0.001, intentionality, *r* = 0.143; *p* < 0.001 and negatively to frequency, *r* = −0.098; *p* = 0.039. Also, severity, intentionally and frequency were positively related: severity-intentionally, *r* = 0.339; *p* < 0.001, severity-frequency *r* = 0.174; *p* < 0.001, intentionally-frequency, *r* = 0.232; *p* < 0.001.

### Measures

#### Forgiveness Seeking Behaviors, Perceived Usefulness Questionnaire (FSB-Q)

As part of this research, we created a questionnaire to assess the usefulness of strategies that may be used by an offender after a relational transgression. As a first step for developing the questionnaire an initial pool of 35 items was generated by the authors based on a review of the literature of forgiveness seeking and relational repair. To assess content validity, the resulting item pool was reviewed by two expert clinical psychologists who were at the time working on transgression and its resolution in the academic setting and in professional practice. The items were reviewed in terms of content relevance, clarity, and ease of understanding. Items that were judged to be redundant or not representative of the content were dropped. Based on the results of the content validation, 21 items from the initial pool were retained. After a pilot study of the first version of the questionnaire in a sample of 157 subjects, some items were modified, resulting in the second version of the questionnaire that is evaluated in this paper.

The 21 items were distributed in four subscales according to the theoretical dimensions. The *Apologies* subscale was comprised of seven items that refer to verbal expressions that communicate to the victim that the offender acknowledges the harm and his/her responsibility for it, and that he/she feels remorseful (e.g., “Showed remorse for the harm caused to me”). The *Restorative action* subscale was made up of six items that describe specific steps taken by the offender to make amends and prevent the offense from happening again (e.g., “Voluntarily took steps to avoid repeating the same actions”). The four items in the subscale of *Caring behaviors* referred to caring and affectionate behaviors not directly linked to the offense (e.g., “Did things that he/she knows I like”). Finally, the *Diverting strategies* subscale was comprised of four items assessing the degree to which the offender tried to minimize his/her responsibility or the harm that has been caused or justify his/her behavior (“Told me that what happened is something normal”). Participants were asked to rate the extent to which they found each statement useful to them in forgiving the offense they had previously recalled with the following prompt: “Rate the following behaviors according to how helpful they were, or they could have been in order to forgive the offender.” They answered on a Likert-type scale ranging from 1 to 5 (1: *Does not help at all* to 5: *It is very helpful*).

#### The Transgression-Related Interpersonal Motivations Inventory (TRIM-18; McCullough et al., 1998, 2006)

The TRIM-18 measures offense-specific forgiveness with three subscales: *Avoidance* (e.g., “I live as if he or she doesn't exist, isn't around”), *Revenge* (e.g., “I'll make him/her pay”), and *Benevolence* (e.g., “Even though his/her actions hurt me, I have goodwill for him/her”). Participants responded on a Likert-type scale ranging from 1 to 5 (1: strongly disagree to 5: strongly agree). The three subscales of the TRIM-18 have good internal consistency (Cronbach's α > 0.87) and test–retest stability (ranging from 0.44 to 0.65). McCullough et al. (1998) validated the questionnaire, as it correlated significantly with other related measures. In our sample, the TRIM-18 subscales showed adequate internal consistency: *Avoidance* (Cronbach's α = 0.868), *Revenge* (Cronbach's α = 0.743), and *Benevolence* (Cronbach's α = 0.772).

#### Offense Characteristics

Regarding the specific offense, a set of questions gathered information about the degree of severity, intentionality, and frequency of the offense. We assessed severity on a Likert-type scale from 1 to 5 (not severe to extremely severe), intentionality on a Likert-type scale from 1 to 5 (not intentional at all to absolutely intentional), and frequency on a 4-point Likert scale (ranging from first time to continuously).

### Data Analysis

Given the ordinal nature of our data (measured on a 5-point Likert scale) and the distribution of item responses, we analyzed the polychoric correlation matrix using Mplus 7.0 latent software (Muthén, and Muthén, 1998–2010) with a robust weighted least squares estimator (WLSMV) (Abad et al., 2011; Brown, 2015). We randomly split the sample in an exploratory (40%, 186 subjects) and a confirmatory (60%, 264 subjects) subsamples.

In the exploratory subsample we run an exploratory factor analysis (EFA) with Geomin rotation and a cut point of 0.4 for factorial weights. Lastly, we run the final model in an AFC on the confirmatory subsample. To evaluate the goodness of fit in the analysis, we used the parsimony correction index root mean square of approximation (RMSEA) and its 90% confidence interval, the Tucker–Lewis index (TLI), the Comparative Fit index (CFI) (Brown, 2015). In the case of the RMSEA, it has been suggested that values < 0.05 constitute a good fit; values in the 0.05 to 0.08 range, an acceptable fit; values in the 0.08 to 0.10 range, a marginal fit; and values > 0.10, a poor fit (Browne and Cudeck, 1992). In the case of the CFI and TLI, Hu and Bentler (1999) propose a cutoff value of 0.95. We also included chi-square model fit information. We explored CFA residuals and modification index in order to detect items with a low fit to the model.

The convergent validity of the subscales with the characteristics of the offense and the TRIM-18 scale was assessed, using the Pearson product-moment correlation coefficient. Type-I error due to multiple tests was corrected via Bonferroni correction (Caperos et al., 2016). Finally, the internal consistency of the subscales was calculated with ordinal approximations of Alpha and Theta based on polychoric correlation matrix (Zumbo et al., 2007).

## Results

An exploratory factor analysis was conducted with ~40% of the sample (*n* = 186). The EFA showed and adequate fit of the data to the theoretical four factor model: RMSEA = 0.081 (0.069–0.094); CFI = 0.981; TLI = 0.970; *X*^2^_(132)_ = 293.612; *p* < 0.001 (Table 1). Items 1, 2, 3, 4, 5, 6, and 7 weighted in the *Apologies* factor, Items 8, 9, 10, 11, 12, 13, and 14 in *Restorative action*, Items 15, 16, 17, and 18 in *Caring behaviors* and Items 19, 20, 21, and 22 in *Diverting strategies*. Correlations among the three first factors are high, *r*_(12)_ = 0.680; *r*_(13)_ = 0.518; *r*_(23)_ = 0.541, while the 4th factor is more independent *r*_(14)_ = 0.073; _r(24)_ = −0.019; *r*_(34)_ = 0.355.

**Table 1:** Factorial weights for the EFA and the CFA.

**Item**	**Subscale**	**EFA**				**CFA**
		**F1**	**F2**	**F3**	**F4**	**Weight (SE)**
Acknowledged that his/her action hurt me	Apologies	**0.756**	0.228	−0.211	−0.011	0.800 (0.028)
Apologized		**0.918**	0.074	−0.114	0.050	0.871 (0.019)
Told me that he/she was sorry and that it will not happen again		**0.867**	−0.024	0.060	0.026	0.813 (0.026)
Admitted his/her responsibility for what happened		**0.855**	0.074	−0.011	−0.086	0.933 (0.012)
Admitted to feeling guilty for what happened		**0.877**	−0.175	0.151	0.037	0.902 (0.013)
Showed remorse for the harm caused to me		**0.876**	−0.016	0.081	−0.069	0.940 (0.010)
Realized the pain he/she had inflicted on me		**0.708**	0.202	−0.001	−0.025	0.896 (0.017)
Allowed me the time I need to forgive	Restorative action	0.075	**0.685**	0.160	0.069	0.872 (0.018)
Didn't talk about it, but his/her actions showed remorse		0.037	**0.877**	−0.020	0.033	0.900 (0.016)
Tried to comfort the hurt feelings		0.357	**0.434**	0.127	0.016	0.836 (0.025)
Voluntarily took steps to avoid repeating the same actions		0.285	**0.515**	0.099	−0.022	0.832 (0.023)
Talked to me about how it affected me so that it won't happen again		0.026	**0.680**	0.318	−0.071	0.867 (0.018)
Made specific changes so my wounds can heal		−0.018	**0.724**	0.248	−0.012	0.915(0.013)
Made an effort to be kind to me	Caring behaviors	0.119	0.215	**0.566**	0.108	0.922 (0.017)
Did things that he/she knows that I like		0.177	0.039	**0.788**	0.035	0.922 (0.013)
Did things for me		−0.033	0.096	**0.915**	−0.042	0.936 (0.012)
Paid more attention to me without directly mentioning the situation		−0.010	0.032	**0.844**	0.045	0.776 (0.028)
Tried to make me feel that what happened was not really that important	Diverting strategies	0.026	−0.086	0.269	**0.735**	0.882 (0.021)
Told me his/her actions were unavoidable		−0.018	0.218	−0.136	**0.966**	0.830 (0.026)
Told me that what happened is something normal		−0.139	0.036	0.036	**0.910**	0.881 (0.020)
Told me it was useless to keep reopening the wound		0.103	−0.116	0.060	**0.770**	0.840 (0.023)

*EFA, factorial weights for the exploratory; CFA, confirmatory factor analysis*.

The final structure was checked in the confirmatory subsample by a CFA (60% of the sample, *n* = 264). The model showed and acceptable to marginal fit, RMSEA = 0.094 (0.086–0.102), CFI = 0.967, TLI = 0.962; *X*^2^_(183)_ = 608.228; *p* < 0.001. Correlations among factors show a similar pattern to the exploratory sample, *r*_(12)_ = 0.866; *r*_(13)_ = 0.682; *r*_(23)_ = 0.785, factor 4 is more independent *r*_(14)_ = 0.095; *r*_(24)_ = 0.080; *r*_(34)_ = 0.342. Results of these analysis can be found in Table 1.

The test's convergent validity was assessed using TRIM-18 and the variables related with the characteristics of the offense (see Table 2).

**Table 2:** Correlations among forgiveness seeking strategies and offense characteristics and forgiveness.

	**Mean**	**SD**	**Perceived Usefulness of Dimensions**			
			**Apologies**	**Restorative action**	**Caring behaviors**	**Diverting strategies**
**TRIM-18 Forgiveness subscales**						
Avoidance	19.0	8.46	*n* = 389; *r* = −0.092; *p* = 0.069	*n* = 385; *r* = −0.080; *p* = 0.118	*n* = 385; *r* = −0.106; *p* = 0.038	*n* = 388; *r* = −0.088; *p* = 0.083
Revenge	6.89	3.41	*n* = 388; *r* = −0.227; *p* < 0.001	*n* = 384; *r* = −0.215; *p* < 0.001	*n* = 384; *r* = −0.105; *p* = 0.040	*n* = 388; *r* = 0.057; *p* = 0.264
Benevolence	20.6	6.79	*n* = 388; *r* = 0.157; *p* = 0.002	*n* = 384; *r* = 0.179; *p* < 0.001	*n* = 384; *r* = 0.266; *p* < 0.001	*n* = 387; *r* = 0.191; *p* < 0.001
**Offense related variables**						
Severity	3.24	1.08	*n* = 447; *r* = −0.004; *p* = 0.939	*n* = 443; *r* = 0.048; *p* = 0.310	*n* = 443; *r* = −0.058; *p* = 0.224	*n* = 446; *r* = −0.156; *p* = 0.001
Intentionality	2.93	1.28	*n* = 446; *r* = −0.185; *p* < 0.001	*n* = 442; *r* = −0.150; *p* = 0.002	*n* = 442; *r* = −0.143; *p* = 0.003	*n* = 445; *r* = −0.118; *p* = 0.013
Offense frequency	1.96	0.93	*n* = 445; *r* = −0.095; *p* = 0.046	*n* = 441; *r* = −0.045; *p* = 0.343	*n* = 442; *r* = −0.044; *p* = 0.360	*n* = 444; *r* = −0.007; *p* = 0.881

*r, Pearson correlation; p, probability value; n, sample size*.

*We applied Bonferroni correction for 24 tests, considering statistically significant correlation with a p-value under p < 0.0021*.

Regarding TRIM-18, the *revenge* subscale presents a significant negative correlation with the *apologies* and *restorative action* subscales, that is, with behaviors that signal assuming responsibility and repairing the harm caused by the offense. The *benevolence* subscale is positively related with the four FSB-Q subscales, *apologies, restorative action, caring behaviors*, and *diverting strategies*, while there are no significant relations between *avoidance* measured by TRIM-18 and the FSB-Q subscales (see Table 2).

Our data show a negative correlation between the *severity* of the offense and the *Diverting strategies* subscale. Also, the *intentionality* of the offense shows a negative relationship with the subscales *apologies, restorative action*, and *caring behaviors*, in this last case close to the significant level considered. There is no relation between the frequency of the offense and the FSB-Q subscales.

We have calculated internal consistency of the subscales in the confirmatory sample finding adequate values of ordinal alpha and Theta, F1: α = 0.960, Θ = 0.952; F2: α = 0.949, Θ = 0.952; F3: α = 0.937, Θ = 0.914; F4: α = 0.918, and Θ = 0.881.

## Discussion

This study is based on McCullough's concept of forgiveness (McCullough et al., 1998) which states that, in addition to apologies, the offender must express a commitment to change and to ensuring that the action will not happen again in the future (Hargrave, 1994; Zechmeister et al., 2004; Fehr and Gelfand, 2010). The first goal of this study was to develop an instrument, the FSB-Q, to assess the victim's perspective of the potential usefulness of the four forgiveness seeking dimensions: apologies, restorative action, relational caring behaviors, and diverting strategies.

The results of the study provide initial evidence that the FSB-Q is a reliable, valid tool to measure the victim's perspective of the utility of a variety of behaviors that offenders might use when they seek forgiveness. These results confirm our proposed four dimensions of forgiveness seeking: in terms of the factorial structure, the EFA supported a four-factor solution.

The results contribute to the study of forgiveness seeking, because they identify and differentiate a range of dimensions in this construct and support aspects that have not been studied with depth in the literature on the seeking of forgiveness. Regarding the first two dimensions, apologies and restorative action, the questionnaire identifies two related but different strategies of seeking forgiveness. Although some questionnaires group verbal responses and behavioral actions in the same dimension (e.g., Morse and Metts, 2011), research has shown that they are needed differentially, depending on the severity and frequency of the offense and the relationship antecedents (Carlisle et al., 2012). Moreover, research shows that changes in behavior are needed to avoid reoffending and improve relationship quality (McNulty, 2011).

The data confirm the existence of a dimension associated with relational caring behaviors. Kelley and Waldron's (2005) questionnaire included items to measure these strategies, but they came under two different dimensions that assessed compensation and nonverbal assurance. We propose that relational caring strategies should be assessed separately, as they do not address the offense directly, but try to soothe and bolster the relationship, and thus are important to fully understanding forgiveness. Further research should be conducted to test their value in contributing to a quality, satisfactory relationship, and their effectiveness for granting forgiveness when they are accompanied by apologies and/or restorative actions.

Our data confirm the relevance of the diverting strategies, also referred as defensive strategies in previous research (Aune et al., 1998; Hodgins and Liebeskind, 2003; Schumann and Orehek, 2017). They are not commonly assessed in the forgiveness seeking literature since these behaviors do not show remorse and are more focused on saving the offender's face but, nevertheless, it is a strategy used by offenders and can be useful to identify persons who might be at risk if they rely mainly on this behaviors in order to forgive. These behaviors are related with what Sheldon and Antony (2018) call pseudo-forgiveness, behaviors that acknowledge the relational offense but ignore or suppress the issue for the sake of the relationship.

Our results show some interesting relationships of forgiveness seeking with offense characteristics.

Regarding *intentionality*, apologies, and restorative action are considered helpful in forgiveness when the offense is viewed as unintentional but, when the offense is perceived as intentional, they are not useful. In these instances, the victim may be acting out of self-protection, feeling that forgiving a purposeful offender would put him or her at risk, and thus attempts at repairing the relationship through apologies and restitution might be perceived as faked (Struthers et al., 2008).

With regard to *severity*, as expected, the more serious the offense, the less useful are strategies based on lessening importance and promoting a change of perspective. Surprisingly, transgression severity is not related to the perception of usefulness of other forgiveness-seeking behaviors (such as apologies or restitution). The reason may be related to the broad range of relational contexts in which the offense occurred in our sample. Less than half the transgressions took place in family or couple relations (out of the total sample, 29% of offenses were committed by a family member and 17% by a partner). Research indicates that forgiveness seeking behaviors are differentially used depending on the level of relationship commitment (Sheldon and Antony, 2018). Further research focusing on specific, close relational settings in which the relationship is likely to continue once forgiveness has been granted, such as couple relations, may reveal that the severity and frequency of the offense is also related with the kind of repair behaviors that are valued by the victim.

In cases of *repeated offenses*, the utility of the different forgiveness-seeking strategies is not related with the offense frequency. This might be linked with the fact that in our sample the variability of the responses in offense frequency is low and the majority of the participants answered the questionnaire regarding low frequency offenses (74.5% reported that it occurred one time or twice).

Focusing on the relationship between forgiveness and the perceived usefulness of forgiveness seeking strategies, we found that the offender's actions are related with the victim's feelings of revenge or benevolence, but no with avoidance. The perception of usefulness of actions such as showing regret and accepting responsibility for the damage is associated with a reduction in the desire for *vengeance*. In other words, both verbal and behavioral responses that directly acknowledge the harm are considered as important to promote this aspect of forgiveness. The desire for revenge is not altered by more indirect behaviors that focus on aspects of the relationship without directly addressing the transgression, or actions with which offenders try to justify their actions or diminish the importance of the transgression.

Regarding *benevolence*, our data show that the four dimensions of forgiveness seeking are associated with a more positive view of the offender. Verbal recognition and specific actions aimed at change are significant, as they express care for the relationship, promote a less blaming view of the offender and present the offense in a less harmful framework. In future research on close relationships, we should analyze whether diverting strategies could be detrimental in the long-term, as they focus on protecting the transgressor self-image over the victim's repair and relationship restoration (Woodyatt and Wenzel, 2013a).

None of the forgiveness seeking behaviors is perceived as useful to reduce the avoidance and distancing from the offender, from the offended perspective. This result might be related again to the non-specificity of the relational context. Research has shown that forgiveness, and therefore reduction of avoidance, is more likely to occur in close relationships where continuity is more likely to occur (Rusbult et al., 1991; Ferrara and Levine, 2009).

The present study has some limitations that should be addressed in future research. First, as the type of relationship with the offender varied widely in our sample, we cannot analyze the importance of this variable in relation with the characteristics of the offense and the dynamics of forgiveness seeking. Second, we did not study the role that the quality of the relation with the offender plays in the perception of usefulness of the forgiveness-seeking behaviors and its relationship with forgiveness. Third, the relationship status (if the relation had ended or not) at the time of the response to the questionnaire was not measured and therefore the relationship with forgiveness-seeking could not be explored.

Despite these limitations, the present findings support the multidimensional perspective of the concept of forgiveness-seeking, and the research provides a valid instrument for measuring the victim's perspective of the relevance of the different strategies for granting forgiveness. The questionnaire can be useful in assessment and interventions related to relational transgressions, as this instrument allows to identify individuals who tend to value forgiveness seeking strategies that might place them at an increased risk of re-offense (i.e., if they only value apologies or diverting strategies). This perspective can be helpful for identifying individuals that forgive the offender when there isn't a behavior change on the offender's part and/or tend to minimize the offense as it has been stated by authors that study the dark side of forgiveness.

Since it pinpoints different behavior patterns of the offender, it will be particularly relevant in identifying which strategies would contribute most to ensuring that the impact of forgiveness on the relationship is positive. Future research will make it possible to determine whether the differential use of the strategies is related to the type of victim-offender relationship and/or with the type of offense, and whether the strategy chosen will have an impact on the victim's wellbeing and the quality of the future relationship that is ultimately forged.

## Data Availability Statement

The datasets presented in this study can be found in online repositories. The names of the repository/repositories and accession number(s) can be found below: figshare at https://figshare.com/s/e2647b5ffa8691cd5881, doi: 10.6084/m9.figshare.13139825.

## Ethics Statement

Ethical review and approval was not required for the study on human participants in accordance with the local legislation and institutional requirements. The patients/participants provided their written informed consent to participate in this study.

## Author Contributions

PM-D: conception, writing – original draft, and supervision. JC: analysis of data, writing – review. MP-U: conception, writing – review, and editing. EG-G and VG: writing – review and editing. MC: conception, writing – original draft, and supervision. All authors contributed to the article and approved the submitted version.

## Conflict of Interest

The authors declare that the research was conducted in the absence of any commercial or financial relationships that could be construed as a potential conflict of interest.

## Footnotes

^1^Information about the data set is available in https://figshare.com/s/e2647b5ffa8691cd5881.

## References

[B1] Abad F. Olea J. Ponsoda V. García C. (2011). Medición en Ciencias Sociales y de la Salud [Measurement in Social and Health Sciences]. Síntesis.

[B2] Afifi W. A. Metts S. (1998). Characteristics and consequences of expectation violations in close relationships. J. Soc. Pers. Relat. 15, 365–392. 10.1177/0265407598153004

[B3] Andrews M. (2000). Forgiveness in context. J. Moral Educ. 29, 75–86. 10.1080/030572400102943

[B4] Ashby H. U. (2003). Being forgiven: toward a thicker description of forgiveness. J. Pastoral Care Couns. 57, 143–152. 10.1177/154230500305700205 12875122

[B5] Aune R. K. Metts S. Hubbard A. S. E. (1998). Managing the outcomes of discovered deception. J. Soc. Psychol. 138, 677–689. 10.1080/00224549809603254 9872063

[B6] Bachman G. F. Guerrero L. K. (2006). Forgiveness, apology, and communicative responses to hurtful events. Commun. Rep. 19, 45–56. 10.1080/08934210600586357

[B7] Bassett R. L. Bassett K. M. Lloyd M.-W. Johnson J. L. (2006). Seeking forgiveness: considering the role of social emotions. J. Psychol. Theol. 34, 111–124. 10.1177/009164710603400201

[B8] Brown T. A. (2015). Confirmatory Factor Analysis for Applied Research. New York, NY: Guilford Publications.

[B9] Browne M. W. Cudeck R. (1992). Alternative ways of assessing model fit. Sociol. Methods Res. 21, 230–258. 10.1177/0049124192021002005

[B10] Caperos J. M. Olmos R. Pardo A. (2016). Inconsistencies in reported p-values in Spanish Journals of Psychology. Methodology 12, 44–51. 10.1027/1614-2241/a000107

[B11] Carlisle R. Tsang J. Ahmad N. Y. Worthington E. L. Witvliet C. Wade N. (2012). Do actions speak louder than words? Differential effects of apology and restitution on behavioral and self-report measures of forgiveness. J. Positive Psychol. 7, 294–305. 10.1080/17439760.2012.690444

[B12] Chiaramello S. Muñoz-Sastre M. T. Mullet E. (2008). Seeking forgiveness: factor structure, and relationships with personality and forgivingness. Pers. Individual Differ. 45, 383–388. 10.1016/j.paid.2008.05.009

[B13] Cordova J. Cautilli J. Simon C. Sabag R. A. (2006). Behavior analysis in forgiveness in Couples Therapy. Int. J. Behav. Consult. Ther. 2, 192–214. 10.1037/h0100776

[B14] Davis J. R. Gold G. J. (2011). An examination of emotional empathy, attributions of stability, and the link between perceived remorse and forgiveness. Pers. Indiv. Differ. 50, 392–397. 10.1016/j.paid.2010.10.031

[B15] Dindia K. (2003). Definition and perspectives on relational maintenance communication, in Maintaining Relationships Through Communication. Relational, Contextual and Cultural Variations, eds Canary D. J. Dainton M. (Mahwah, NJ: Lawrence Erlbaum Associates), 1–30.

[B16] Dindia K. Baxter L. (1987). Strategies for maintaining and repairing marital relationships. J. Pers. Soc. Relat. 4, 143–158. 10.1177/0265407587042003

[B17] Emmers T. M. Canary D. J. (1996). The effect of uncertainty reducing strategies on young couples' relational repair and intimacy. Commun. Q. 44, 166–182. 10.1080/01463379609370008

[B18] Exline J. J. Baumeister R. F. (2001). Expressing forgiveness and repentance. Benefits and barriers, in Forgiveness: Theory, Research and Practice, eds McCullough M. E. Pagament K. I Thorensen C. E. (New York, NY: The Guilford Press), 133–155.

[B19] Exline J. J. Worthington E. L. Hill P. McCullough M. E. (2003). Forgiveness and justice: a research agenda for social and personality psychology. Personality and Social Psychology Review. 7, 337–348. 10.1207/S15327957PSPR0704_06 14633470

[B20] Fehr R. Gelfand M. J. (2010). When apologies work: How matching apolgy components to victim's self construals facilitates forgiveness. Organ. Behav. Hum. Decis. Process. 113, 37–50. 10.1016/j.obhdp.2010.04.002

[B21] Ferrara M. Levine T. R. (2009). Can't live with them or can't live without them?: the effects of betrayal on relational outcomes in college dating relationships. Commun. Q. 57, 187–204. 10.1080/01463370902881734

[B22] Fincham F. D. Hall J. Beach S. R. (2006). Forgiveness in marriage: current status and future directions. Fam. Relat. 55, 415–422. 10.1111/j.1741-3729.2005.callf.x-i1

[B23] Fincham F. D. Jackson H. Beach S. R. H. (2005). Transgression severity and forgiveness: Different moderators for objective and subjective severity. J. Soc. Clin. Psychol. 24, 860–875. 10.1521/jscp.2005.24.6.860

[B24] Gold G. J. Weiner B. (2000). Remorse, confession, group identity, and expectancies about repeating a transgression. Basic Appl. Soc. Psychol. 22, 291–300. 10.1207/S15324834BASP2204_3

[B25] Gordon K. C. Amer A. Lenger K. A. Brem M. J. Baucom D. H. Snyder D. K. (2020). Forgiveness and the dark side of intimate relationships, in Handbook of Forgiveness, 2nd edn., eds Worthington E. L. Wade N. G. (New York, NY: Routledge), 153–177.

[B26] Gordon K. C. Baucomb D. H. (2003). Forgiveness and marriage: preliminary support for a measure base don a model of recovery from a marital betrayal. Am. J. Fam. Ther. 31, 179–199. 10.1080/01926180301115

[B27] Gordon K. C. Baucomb D. H. Snyder D. K. (2004). An integrative intervention for promoting recovery from extramarital affairs. J. Marital Fam. Ther. 30, 213–231. 10.1111/j.1752-0606.2004.tb01235.x 15114949

[B28] Gracyalny M. L. Jackson D. C. Guerrero L. K. (2008). A second look Forgiveness and communicative responses to hurtful events, in Paper presented to the National Communication Association Convention (San Diego, CA).

[B29] Green J. D. Reid C. A. Coy A. E. Hedgebeth M. V. Kneuer M. A. (2020). An interdepende analysis of forgiveness, amends, and relational repair in family and work relationships, in Handbook of Forgiveness, 2nd edn, eds Worthington E. L. Wade N. G. (New York, NY: Routledge), 131–141.

[B30] Guerrero L. Andersen P. Afifi W. (2014). Close Encounters: Communication in Relationships, 4th edn. Los Angeles, CA: Sage.

[B31] Gunderson P. R. Ferrari J. R. (2008). Forgiveness of sexual cheating in romantic relationships: effects of discovery method, frequency of offense, and presence of apology. North Am. J. Psychol. 10, 1–14.

[B32] Hannon P. A. Rusbult C. E. Finkel E. J. Kumashiro M. (2010). In the wake of betrayal: amends, forgiveness, and the resolution of betrayal. Pers. Relat. 17, 253–278. 10.1111/j.1475-6811.2010.01275.x

[B33] Hargrave T. D. (1994). Families and forgiveness: a theoretical and therapeutic framework. Fam. J. 2, 339–348. 10.1177/1066480794024007

[B34] Hargrave T. D. Zasowski N. E. (2017). Families and Forgiveness: Healing Wounds in the Intergenerational Family. New York, NY: Routledge.

[B35] Hodgins H. S. Liebeskind E. (2003). Apology versus defense: antecedents and consequences. J. Exp. Soc. Psychol. 39, 297–316. 10.1016/S0022-1031(03)00024-6

[B36] Hu L. T. Bentler P. M. (1999). Cutoff criteria for fit indexes in covariance structure analysis: Conventional criteria versus new alternatives. Struct. Equation Model. Multidiscipl. J. 6, 1–55. 10.1080/10705519909540118

[B37] Karremans J. C. Van Lange P. A. M. (2004). Back to caring after being hurt: the role of forgiveness. Eur. J. Soc. Psychol. 34, 207–227. 10.1002/ejsp.192

[B38] Kelley D. (1998). The communication of forgiveness. Commun. Stud. 49, 255–271. 10.1080/10510979809368535

[B39] Kelley D. L. Waldron V. R. (2005). An investigation of forgiveness-seeking communication and relational outcomes. Commun. Q. 53, 339–358. 10.1080/01463370500101097

[B40] Kloeber D. N. Waldron V. R. (2017). Expressing and suppressing conditional forgiveness in serious romantic relationships, in Communicating Interpersonal Conflict in Close Relationships: Contexts, Challenges and Opportunities, ed. Samp J. A. (Routledge), 250–267.

[B41] Lazare A. (2004). On Apology. New York, NY: Oxford University Press.

[B42] Lewis J. T. Parra T. R. Cohen R. (2015). Apologies in close relationships: a review of theory and research. J. Fam. Theory Rev. 7, 47–61. 10.1111/jftr.12060

[B43] Luchies L. B. Finkel E. J. McNulty J. K. Kumashiro M. (2010). The doormat effect: when forgiving erodes self-respect and self-concept clarity. J. Pers. Soc. Psychol. 98, 734–749. 10.1037/a0017838 20438221

[B44] McCullough M. Rachal K. Sandage S. Worthington E. Brown S. Hight T. (1998). Interpersonal forgiving in close relationships: II. Theoretical elaboration and measurement. J. Pers. Soc. Psychol. 75, 1586–1603. 10.1037/0022-3514.75.6.1586 9914668

[B45] McCullough M. E. (2000). Forgiveness as human strength: theory, measurement, and links to well-being. J. Soc. Clin. Psychol. 19, 43–55. 10.1521/jscp.2000.19.1.43

[B46] McCullough M. E. (2008). Beyond Revenge: The Evolution of the Forgiveness Instinct. San Francisco, CA: Jossey-Bass.

[B47] McCullough M. E. Root L. M. Cohen A. D. (2006). Writing about the benefits of an interpersonal transgression facilitates forgiveness. J. Consult. Clin. Psychol. 74, 887–897. 10.1037/0022-006X.74.5.887 17032093

[B48] McLaughlin M. L. Cody M. J. O'Hair H. D. (1983). The management of failure events: some contextual determinants of accounting behavior. Hum. Commun. Res. 9, 208–224. 10.1111/j.1468-2958.1983.tb00695.x

[B49] McNulty J. K. (2008). Forgiveness in marriage: putting the benefits into context. J. Fam. Psychol. 22, 171–175. 10.1037/0893-3200.22.1.171 18266545

[B50] McNulty J. K. (2011). The dark side of forgiveness: the tendency to forgive predicts continued psychological and physical aggression in marriage. Pers. Soc. Psychol. Bull. 37, 770–783. 10.1177/0146167211407077 21558557 PMC4112745

[B51] McNulty J.K. (2020). Highlighting the dark side of forgiveness and the need for a contextual approach, in Handbook of Forgiveness, 2nd Edn., eds Worhtington E. L. Wade N. G. (New York, NY: Rouledge), 33–43.

[B52] Merolla A. J. (2008). Communicating forgiveness in friendships and dating relationships. Commun. Stud. 59,114–131. 10.1080/10510970802062428

[B53] Merolla A. J. Zhang S. (2011). In the wake of transgressions: examining forgiveness communication in personal relationships. Pers. Relat. 18, 79–95. 10.1111/j.1475-6811.2010.01323.x

[B54] Metts S. Cupach W. R. (2007). Responses to relational transgressions: hurt, anger, and sometimes forgiveness, in The Dark Side of Interpersonal Communication, eds Spitzberg B. H. Cupach W. R. (Mahwah, NJ: Lawrence Erlbaum Associates), 243–274.

[B55] Morse C. R. Metts S. (2011). Situational and communicative predictors of forgiveness following a relational transgression. West. J. Commun. 75, 239–258. 10.1080/10570314.2011.571652

[B56] Muthén L. K. Muthén B. O. (1998–2010). Mplus User's Guide, 6th edn. Los Angeles, CA: Muthén and Muthén.

[B57] Ohbuchi K. Kameda M. Agarie N. (1989). Apology as aggression control: its role in mediating appraisal of and responses to harm. J. Pers. Soc. Psychol. 56, 219–227. 10.1037/0022-3514.56.2.219 2926625

[B58] Ohbuchi K. Sato D. (2001). Children's reactions to mitigating accounts: apologies, excuses and intentionality of harm. J. Soc. Psychol. 134, 5–17. 10.1080/00224545.1994.9710877

[B59] Pansera C. (2009). “If You Don't ‘Get It', It Doesn't Count': Conveying Responsiveness in Attempts to Seek Forgiveness Within Romantic Relationships. Master's Thesis, University of Waterloo, Ontario. Available online at: https://uwspace.uwaterloo.ca/bitstream/handle/10012/4296/Pansera_Carolina.pdf?seqquence=1

[B60] Pansera C. (2012). An Experimental Study of the Effects of Partners'offers of Amends and Expressions of Responsiveness on Forgiveness for Real-Life Transgressions in Romantic Relationships. Unpublished Doctoral Dissertation, University of Waterloo, Ontario. Available online at: https://uwspace.uwaterloo.ca/bitstream/handle/10012/4296/Pansera_Carolina.pdf?sequence=1

[B61] Pansera C. La Guardia J. (2012). The role of sincere amends and perceived partner responsiveness in forgiveness. Pers. Relat. 19, 696–711. 10.1111/j.1475-6811.2011.01386.x

[B62] Rourke J. (2007). Towards the Development of a Questionnaire of Post Transgression Motives and Behaviours. Unpublished Master Dissertation, Brock University, St. Catharines, ON.

[B63] Rusbult C. E. Hannon P. A. Stocker S. L. Finkel E. J. (2005). Forgiveness and relational repair, in Handbook of Forgiveness, ed Worthington E. L. (New York, NY: Routledge), 185–205.

[B64] Rusbult C. E. Verette J. Whitney G. A. Slovik L. F. Lipkus I. (1991). Accommodation processes in close relationships: theory and preliminary empirical evidence. J. Pers. Soc. Psychol. 60, 53–78. 10.1037/0022-3514.60.1.53

[B65] Sandage S. J. Worthington E. L. Hight T. L. Berry W. (2000). Seeking forgiveness: theoretical context and an initial empirical study. J. Psychol. Theol. 28, 21–35. 10.1177/009164710002800102

[B66] Schumann K. Orehek E. (2017). Avoidant and defensive: adult attachment and quality of apologies. J. Soc. Pers. Relat. 36, 809–833. 10.31234/osf.io/au8g4

[B67] Sheldon P. Antony M. G. (2018). Forgive and forget: a typology of transgressions and forgiveness strategies in married and dating relationships. West. J. Commun. 83, 232–251, 10.1080/10570314.2018.1504981

[B68] Slocum D. Allan A. Allan M. M. (2011a). An emerging theory of apology. Aust. J. Psychol. 63, 83–92. 10.1111/j.1742-9536.2011.00013.x

[B69] Slocum D. Mckillop D. R. Allan A. Allan M. M. Drake D. G. (2011b). Apology and acceptance in intimate partner relationships, in Interpersonal Acceptance and Rejection: Social, Emotional and Educational Contexts, eds Kourkoutas E. Erkman F. (Irvine, CA: Brown Walker Press), 175–181.

[B70] Struthers C. W. Eaton J. Santelli A. G. Uchiyama M. Shirvani N. (2008). The effects of attributions of intent and apology on forgiveness: when saying sorry may not help the story. J. Exp. Soc. Psychol. 44, 983–992. 10.1016/j.jesp.2008.02.00628848484

[B71] Waldron V. R. Kelley D. L. (2005). Forgiving communication as a response to relational transgressions. J. Soc. Pers. Relat. 22, 723–742. 10.1177/0265407505056445

[B72] Waldron V. R. Kelley D. L. (2008). Communicating Forgiveness. Los Angeles, CA: Sage.

[B73] Wohl M. McLaughlin K. J. (2014). Self-forgiveness: the good, the bad, and the ugly. Soc. Pers. Psychol. Compass 8, 422–435. 10.1111/spc3.12119

[B74] Woodyatt L. Wenzel M. (2013a). Self-forgiveness and restoration of an offender following an interpersonal transgression. J. Soc. Clin. Psychol. 32, 225–259. 10.1521/jscp.2013.32.2.225

[B75] Woodyatt L. Wenzel M. (2013b). The psychological immune response in the face of transgressions: pseudo self-forgiveness and threat to belonging. J. Exp. Soc. Psychol. 49, 951–958. 10.1016/j.jesp.2013.05.016

[B76] Zechmeister J. S. Garcia S. Romero C. Vas S. N. (2004). Don't apologize unless you mean it: a laboratory investigation on forgiveness and retaliation. J. Soc. Clin. Psychol. 23, 532–564. 10.1521/jscp.23.4.532.40309

[B77] Zumbo B. D. Gadermann H. M. Zeisser C. (2007). Ordinal versions of coefficients alpha and theta for Likert rating scales. J. Mod. Appl. Stat. Methods 6, 21–29. 10.22237/jmasm/1177992180

